# *HLXB9* Gene Expression, and Nuclear Location during *In Vitro* Neuronal Differentiation in the SK-N-BE Neuroblastoma Cell Line

**DOI:** 10.1371/journal.pone.0105481

**Published:** 2014-08-19

**Authors:** Claudia Giovanna Leotta, Concetta Federico, Maria Violetta Brundo, Sabrina Tosi, Salvatore Saccone

**Affiliations:** 1 Dipartimento di Scienze Biologiche, Geologiche e Ambientali, Sezione di Biologia Animale, University of Catania, Catania, Italy; 2 Leukaemia and Chromosome Research Laboratory, Division of Biosciences, Brunel University, London, United Kingdom; University of Navarra, Spain

## Abstract

Different parts of the genome occupy specific compartments of the cell nucleus based on the gene content and the transcriptional activity. An example of this is the altered nuclear positioning of the *HLXB9* gene in leukaemia cells observed in association with its over-expression. This phenomenon was attributed to the presence of a chromosomal translocation with breakpoint proximal to the *HLXB9* gene. Before becoming an interesting gene in cancer biology, *HLXB9* was studied as a developmental gene. This homeobox gene is also known as *MNX1* (motor neuron and pancreas homeobox 1) and it is relevant for both motor neuronal and pancreatic beta cells development. A spectrum of mutations in this gene are causative of sacral agenesis and more broadly, of what is known as the Currarino Syndrome, a constitutional autosomal dominant disorder. Experimental work on animal models has shown that *HLXB9* has an essential role in motor neuronal differentiation. Here we present data to show that, upon treatment with retinoic acid, the *HLXB9* gene becomes over-expressed during the early stages of neuronal differentiation and that this corresponds to a reposition of the gene in the nucleus. More precisely, we used the SK-N-BE human neuroblastoma cell line as an *in* *vitro* model and we demonstrated a transient transcription of *HLXB9* at the 4^th^ and 5^th^ days of differentiation that corresponded to the presence, predominantly in the cell nuclei, of the encoded protein HB9. The nuclear positioning of the *HLXB9* gene was monitored at different stages: a peripheral location was noted in the proliferating cells whereas a more internal position was noted during differentiation, that is while *HLXB9* was transcriptionally active. Our findings suggest that *HLXB9* can be considered a marker of early neuronal differentiation, possibly involving chromatin remodeling pathways.

## Introduction

It is well know that the human genome is distributed in organized structures that occupy specific areas of the nucleus named chromosome territories [1]. Several studies have shown that different parts of the genome occupy specific compartments of the cell nucleus based on their gene content, with gene rich regions positioned towards the nuclear interior and gene poor regions positioned towards the periphery of the nucleus [1]–[6]. The maintenance of higher order chromatin structure is crucial for the maintenance of nuclear health and alterations of this equilibrium are emerging factors in human diseases, including cancer [7]–[12]. Many studies also demonstrated that the chromatin arrangement in the nucleus has a correlation with cellular functions, including differentiation [13]–[19], and that gene distribution in different regions of the nucleus is also associated with transcriptional activity [20]–[26]. For instance, an altered nuclear positioning of the *HLXB9* gene was shown in leukaemia cells in association with gene over-expression, a phenomenon that was attributed to the presence of a chromosomal translocation with breakpoint proximal to the *HLXB9* gene [12].

The *HLXB9* gene, also known as *MNX1* (motor neuron and pancreas homeobox 1), is located on chromosome 7q36.3 and belongs to the family of EHG homeobox genes which includes also *EN1*, *EN2*, *GBX1* and *GBX2* [27], [28]. *HLXB9* is a gene of 12,801 bp, is composed of 3 exons and codes for a transcription factor, HB9, formed by 401 aminoacids [29]. HB9 contains a homeodomain, preceded by a highly conserved region of 82 amino acids (159–241) and a region of polyalanine that expands from residue 121 to residue 134 in exon 1 [30].

*HLXB9* was identified as a locus involved in the autosomal dominant Currarino Syndrome, also known as Hereditary Sacral Agenesis (HSA) syndrome: impaired function of the *HLXB9* gene generates a disorder characterized by rectal and uro-genital malformations and sacral agenesis. Malformations observed in the Currarino syndrome probably reflect disturbances in secondary neurulation, a process that occurs in the early stages of development [31].

The *HLXB9* gene is also involved in development of pancreatic beta cell [29], [32] and motor neuronal cells with an essential role in motor neuronal differentiation. Specific expression of *HLXB9* in pancreatic beta cells is associated with the conserved function in beta cells maturation. The gene is expressed in zebrafish and mice during two different stages of pancreas development. Before the embryonic stage of morphogenesis, *HLXB9* is expressed in the pancreatic endoderm, but with morphogenesis this gene is down-regulated and subsequently reactivated during beta cells differentiation. This makes the *HLXB9* gene an early specific marker of differentiation of pancreatic cells and suggests that this gene is linked to the initial steps of beta cells specification [33].

RNA *in* *situ* hybridization experiments on the amphioxus embryo have revealed that *AmphiMnx* (orthologue of the human *HLXB9* gene) has a dynamic pattern of expression in the neuroectoderm and the mesoderm. The gene transcript is detected ten hours after fertilization and decreases in the following hours [34].

Moreover, *HLXB9* is expressed during motor neuron differentiation and it is part of the regulatory system for this process [35], [36]. Genetic studies in the mouse highlight the importance of *HLXB9* in the consolidation and maintenance of motor neurons identity suggesting that *HLXB9* is to be considered a marker for the correct development of spinal neurons. In fact, mice lacking functional *HLXB9* generate a correct number of motor neurons but show dramatic changes in their program of differentiation, such as: abnormalities in the pattern of migration, errors in the motor axons projection and innervation defects in some target muscles [35]. Moreover, studies performed on neuroepithelial stem cells, showed an increase in the levels of *HLXB9* transcripts in motoneuronal phenotype [37].

Neuroblastoma cell lines have been used extensively as *in* *vitro* models for studies on neuronal development including proliferation, differentiation and growth [38], [39], [40]. In particular, it has been shown that it is possible to obtain neuron-like cells after treatment with retinoic acid [38]. Being *HLXB9* gene involved in motor neuron identity, we wanted to investigate its expression levels during neuronal cell differentiation, using the human neuroblastoma cell line SK-N-BE as *in* *vitro* model, and its nuclear location to point out a possible correlation between the expression of *HLXB9* and the chromatin re-organization, as previously shown for this gene in leukaemic cells [12].

Here we demonstrate, for the first time, that *HLXB9* is expressed in a specific and restricted period during *in* *vitro* human neuronal differentiation in the SK-N-BE neuroblastoma cell line, and that *HLXB9* expression is associated with a change of its radial nuclear location, indicating *HLXB9* as an early specific marker of neuronal cell differentiation, a process that implies a remodelling of the chromatin organization.

## Materials and Methods

### Cell cultures

Human neuroblastoma cell line SK-N-BE [38] grows in RPMI 1640 supplemented with fetal bovine serum (FBS) to a final concentration of 10%, 1% antibiotic Penicillin/Streptomycin (P/S) and 1% L-Glutamine at 37°C, with 5% CO_2_ [38]. SK-N-BE cells were kindly gifted by Professor Della Valle from Department of Genetics and Microbiology, University of Pavia, Italy [41].

Human myeloma U266 cells were kindly provided by Dr. Daniele Tibullo, Department of Clinical and Molecular Biomedicine, University of Catania, Catania, Italy [42], purchased from ATCC (code number TIB-196). Cells were maintained in RPMI 1640 medium supplemented with fetal bovine serum (FBS) to a final concentration of 10%, 1% antibiotic Penicillin/Streptomycin (P/S) and 1% L-Glutamine at 37°C, with 5% CO_2_ [43], [44].

Human liver cancer HepG2 cell line were kindly provided by Professor Fabrizio Palitti, Department of Ecological and Biological Sciences, University of Tuscia, Viterbo, Italy [45]. Cells were grown in Dulbecco’s minimal essential medium (DMEM) supplemented with 15% fetal bovine serum, 1% antibiotic Penicillin/Streptomycin (P/S) and 1% L-Glutamine at 37°C, with 5% CO_2_ [46].

SK-N-BE cells were differentiated in neuronal-like cells after treatment with retinoic acid (10 µM). Retinoic acid was added to the culture medium every 72 hours (day 0, 3, 6, 9) to obtain fully differentiated cells at the 12th day of treatment [47], [48]. U266 and HepG2 cells were used as controls and treated with retinoic acid (10 µM) for six days.

### *In situ* hybridization on chromosomes and nuclei

To obtain metaphase chromosomes we added colcemid 0.05 µg/ml to the cell cultures for 1 hour before harvesting. Then, cells were harvested in hypotonic solution (KCl 0.075 M) and fixed with methanol-acetic acid (3∶1). Interphase nuclei were prepared using a protocol to preserve the 3D chromatin structure, as previously described [49]. Briefly, cells were fixed in freshly made 4% paraformaldehyde in PBS for 10 min, washed in PBS, incubated in 0.5% Triton X-100 in PBS for 10 min, and equilibrated in 20% glycerol in PBS for 30 min. Cells were then frozen in liquid nitrogen and thawed at room temperature. After a further wash in PBS, cells were incubated for 5 min in 0.1 N HCl. Subsequently, fluorescence *in* *situ* hybridizations was performed with PAC RPCI-5-1121A15 probe containing *HLXB9* gene. DNA probes were extracted using a commercial kit (Qiagen, Milan, Italy), biotin-labeled by nick translation (Roche, Mannheim, Germany) and hybridized as previously described [6]. Detection of hybridized probe was performed using fluorescein-conjugated avidin.

### Statistical analysis

Images of the hybridized nuclei were captured using MacProbe v4.3 software (Applied Imaging, Newcastle, UK), and radial nuclear location of *HLXB9* gene was obtained using the two-dimensional (2D) analysis as previously described [6]. Briefly, the radial nuclear position was first assigned to each hybridization signal in each cell nucleus as a ratio of the nuclear radius (0 and 1 indicate the centre and the periphery of the nucleus, respectively) using a dedicated computer software developed in our lab at the University of Catania [6]. The assessment of each radial nuclear location was based on the analysis of a minimum of 300 nuclei per experiment. At least two different experiments per each examined period (proliferative cells, and cells after 2, 4, 6, and 12 days of treatment with retinoic acid) were performed. Nuclear location was defined as the median value ± confidence interval (C.I.): median values lower than 0.65 indicate loci located more internally in the nuclei. The statistical analyses were carried out using Microsoft Excel and StatView softwares.

### Expression analysis

To analyze gene expression in SK-N-BE cells, RNA was extracted every day from 0 (start) to 12 days (fully differentiated cells), after retinoic acid induction, using TRI Reagent (Sigma-Aldrich, St. Louis, USA) and used in Real Time PCR experiments (StepOne instrument from Applied Biosystems) with primers specifically selected for target and control genes (Sigma-Aldrich, St. Louis, USA) (Table 1). To analyze *HLXB9* expression in the control cell lines U266 and HepG2, RNA was extracted at day 0, 4 and 6 after retinoic acid induction with the same conditions used for SK-N-BE cells.

**Table 1:** Primers used in the present study.

Human Gene	Size	Nucleotide sequence (5′-3′)	
*HLXB9*	137 bp	Forward	AACCTCCTGGGGAAGTGC
		Reverse	GGTGAGCATGAGCGAGGT
*ACTB*	131 bp	Forward	GACGACATGGAGAAAATCTG
		Reverse	ATGATCTGGGTCATCTTCTC
*GAP-43*	76 bp	Forward	GAGGAAAAATCTTCAGAGACC
		Reverse	AACCCTTGAAATCCAGAAAG
*MYCN*	187 bp	Forward	GAGCGATTCAGATGATGAAG
		Reverse	TCGTTTGAGGATCAGCTC
*MAPT*	190 bp	Forward	CTGGTTTGGGTACAGTTAAAG
		Reverse	AGATCCCTTCAACTTAGGAG

Real Time PCR was performed according to the manufacturer instructions with specific annealing temperature and experiments were repeated at least three times with statistical analyses for each individual experimental set. A value of P<0.05 was considered statistically significant. Data were represented as mean ± standard error of the mean (SEM). Data on *HLXB9*, *GAP43*, *MAPT* and *MYCN* were analyzed using the Ct value. This is normalized with Ct value of the endogenous control *ACTB* obtaining the ΔCt. Normalizing the ΔCt of the sample of interest with that of the calibrator, we get the ΔΔCt. Finally, to determine the relative concentration of the target gene in the sample of interest we applied 2^−ΔΔCt^ formula.

### Immunolocalization

Indirect immunofluorescence experiments were performed on SK-N-BE cells using anti HB9 antibody (Sigma–Aldrich, 1∶100 dilution) at day 0, 4, 5 and 6 after retinoic acid treatment, and anti β-tubulin antibody (Sigma–Aldrich, 1∶100 dilution) at day 0 and 12. Cells were fixed with 4% paraformaldehyde for 20 min at room temperature. Fixed cells were washed with PBS and incubated 15 min in PBS containing 0.5% Triton X-100, and then for 20 min in blocking solution. The cells were then incubated with specific primary antibody overnight at 37°C. After further PBS washes, the cells were incubated for 1 hour with anti-rabbit secondary antibody conjugated with FITC (Invitrogen, 1∶100) at 37°C. Photographs were taken under a Zeiss LSM 700 microscope.

## Results

### Assessment of differentiation in SK-N-BE cells

**Figure 1:**
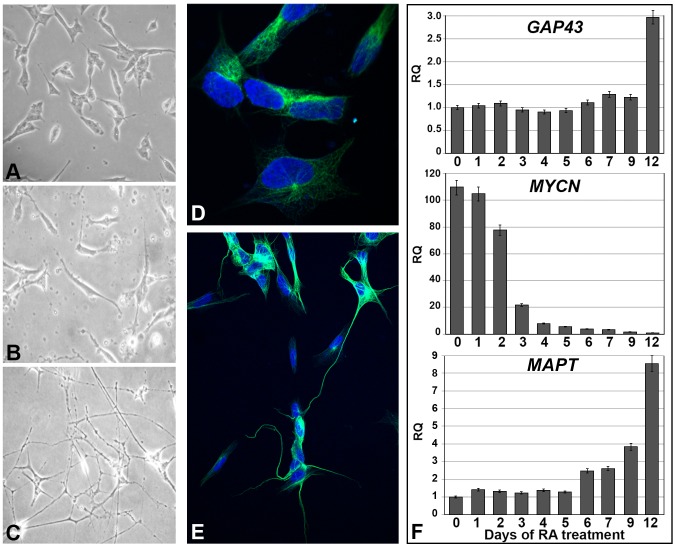
Morphological changes and expression analysis of neuronal differentiation markers in SK-N-BE cells. (A, B, C) Morphology of the SK-N-BE cells in proliferating stage (A), and at 6^th^ (B) and 12^th^ (C) day after treatment with retinoic acid. The cell body from relatively round shape (A) becomes more elongated (B) and cells eventually form dendridic-like extensions (C). Images were obtained using the inverted microscope Olympus CK40 (200x magnification). (D, and E) Dendridic-like extensions are clearly visible by indirect immunofluorescence experiments to detect β-tubulin protein in SK-N-BE cells at the 12^th^ day after treatment with retinoic acid (E) respect to the proliferating stage (D). These images were obtained using the Zeiss LSM700 Confocal Laser Scanning Microscopy. (F) Histograms showing the expression profile of *GAP-43, MYCN,* and *MAPT* genes, three control genes differently expressed during cell differentiation, reaching the peak of expression on the twelfth day (*GAP-43* and *MAPT*), or on the early days (*MYCN*), when SK-N-BE treated with retinoic acid are differentiated in a neuron-like form. 0 indicates proliferating cells before treatment with retinoic acid.

SK-N-BE cells treated with retinoic acid start differentiating into neuronal-like cells. After 12 days of *in* *vitro* treatment, cells are fully differentiated. During differentiation, cells stop proliferating and undergo multiple morphological modifications that involve loss of round shape and development of cell protrusions to resemble dendritic formations (Figure 1 A, B, C, D and E). Real-time PCR experiments were performed on RNA samples extracted from SK-N-BE cells on a daily basis during differentiation, and specific markers to confirm neuronal differentiation were used. These experiments showed expression of *GAP-43* and *MAPT* gene throughout the 12 days, with a dramatic increase at day 12. Conversely, *MYCN* expression was higher at the initial stages of retinoic acid treatment and decreases with time during differentiation (Figure 1F).

### *HLXB9* gene expression in SK-N-BE

**Figure 2:**
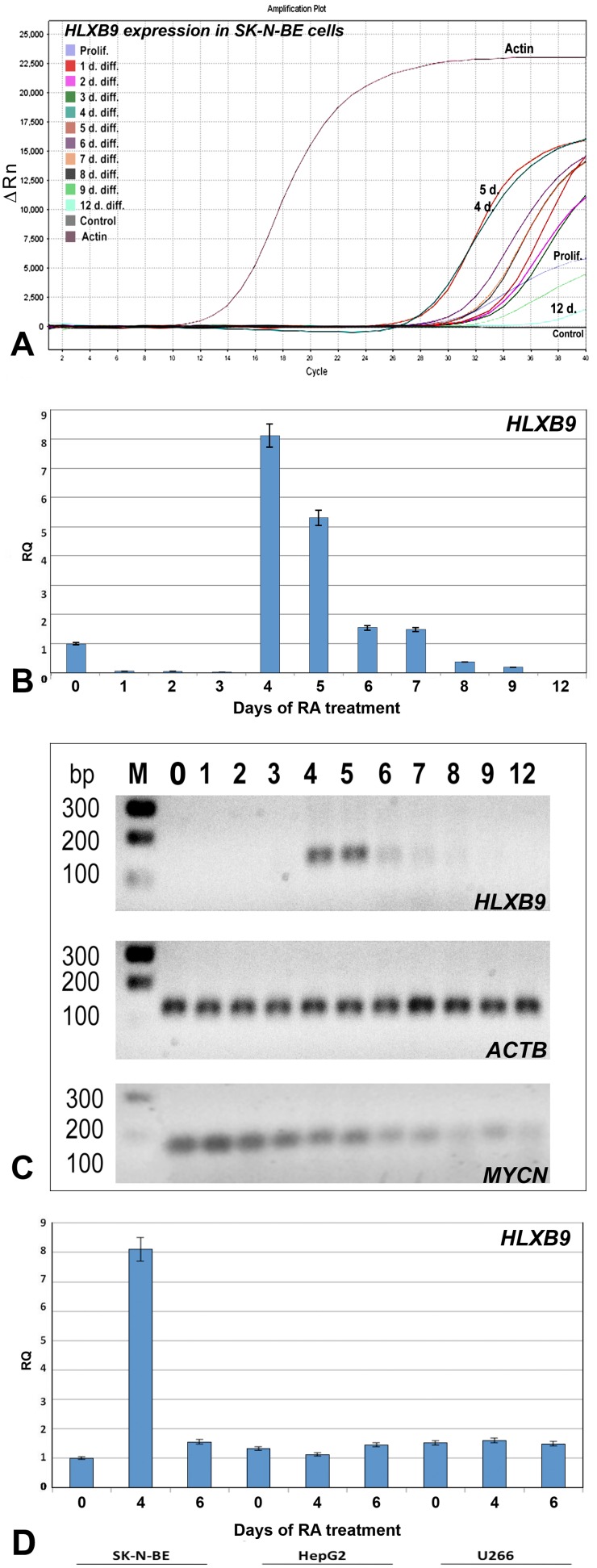
*HLXB9* expression in SK-N-BE. (A) Amplification plot of Real time PCR experiments. Data show *HLXB9* expression in SK-N-BE cells at different days after retinoic acid treatment in comparison with expression of *ACTB* (Actin-B) used as a control. (B) Histogram generated with values of 2^−ΔΔCt^. Samples are on x-axis and fluorescence emission is on y-axis. The increase of *HLXB9* expression in SK-N-BE cells is noted dramatically on the fourth and fifth day of differentiation. Subsequently, *HLXB9* expression decreases until disappearing at day 12. (C) Electrophoresis of the Real time PCR fragments obtained with the specific primers for *HLXB9* and for the control gene *ACTB*. Electrophoresis of *MYCN* Real time PCR fragments was also shown. In B, and C “0” indicates proliferating cells before treatment with retinoic acid. D) Histogram generated with values of 2^−ΔΔCt^. Samples are on x-axis and fluorescence emission is on y-axis. There isn’t a significant increase of *HLXB9* gene expression in U266 and HepG2 cells, when cells are treated with retinoic acid. Data in the graphs B, and D are represented as mean ± standard error of the mean (SEM).

Real-time PCR showed the presence of *HLXB9* mRNA mainly in the fourth and fifth day of differentiation, then undergoing a significant decrease on the sixth day and disappearing from seventh to twelfth day of differentiation. Analysis of data obtained from 2^−ΔΔCt^ calculation enabled us to quantify the increase of *HLXB9* expression, with an eightfold increase at the fourth day and a fivefold increase at the fifth day compared to proliferating cells (Figure 2B).

To confirm that our observations are specific for the SK-N-BE cell line and that retinoic acid itself does not influence *HLXB9* gene expression we used two other different cell lines as control: U266 and HepG2 cells, derived from myeloma and liver cancer respectively. These cell lines, exposed to retinoic acid for six days, did not show an increase in *HLXB9* expression (Figure 2D).

### *HB9* immunodetection

**Figure 3:**
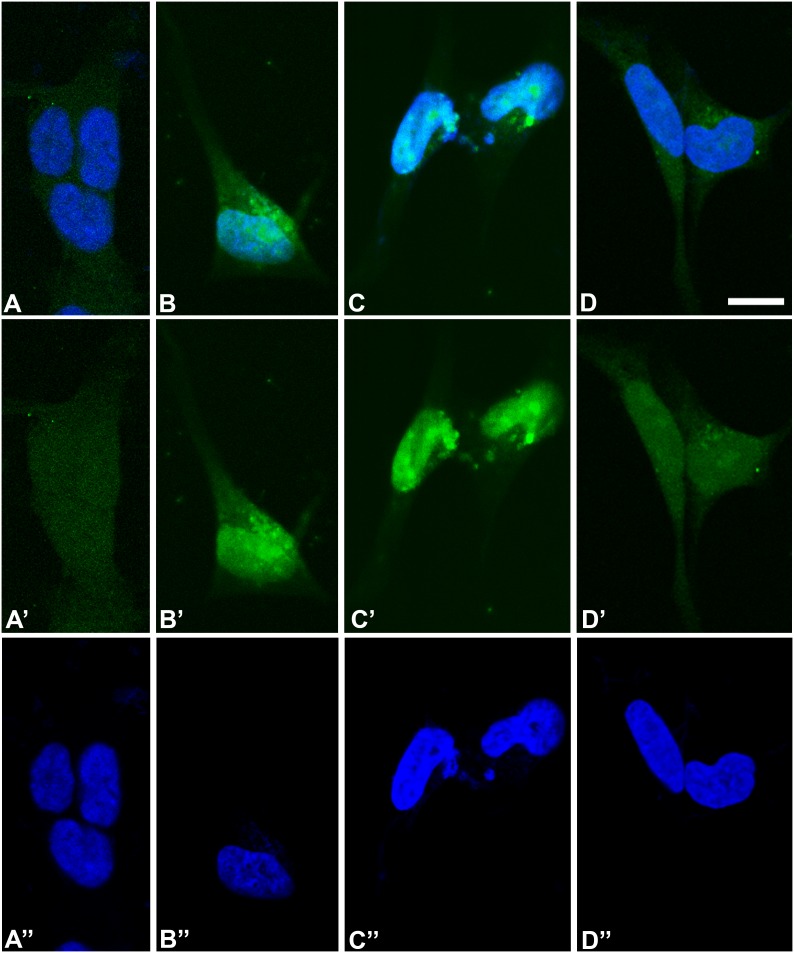
Indirect immunofluorescence to detect HB9 protein in SK-N-BE cells. A, B, C, and D show SK-N-BE cells at proliferative stage (day 0) and at 4^th^ day, 5^th^ day, and 6^th^ day after acid retinoic induction respectively. HB9 protein (green signal) is visible in both nucleus and cytoplasm at the fourth and fifth days of differentiation (B, C). A very faint staining is also noted in the cytoplasm of cells at proliferating stage (A) and in the cytoplasm of cells at the sixth day of differentiation (D). A, B, C and D are the merge images of A’, B’, C’, D’, representing immunofluorescence staining only, and A”, B”, C”, D”, representing the DAPI staining of the nucleus only in blue. Scale bar is 10 µm.

In agreement with the Real time PCR data showing *HLXB9* gene expression, the HB9 protein was detected in the SK-N-BE cells at fourth and fifth day of differentiation by immunofluorescence with specific antibody. HB9 immunostaining was detected in both nuclei and cytoplasm of cells at the fourth and fifth day of differentiation, with more intense staining in the nucleus than in the cytoplasm. This preferential nuclear location is compatible with the function of HB9 protein as transcription factor. Cells at sixth day of differentiation showed a decrease of the HB9 presence in the nucleus (Figure 3).

### Radial nuclear location of the *HLXB9* gene

**Figure 4:**
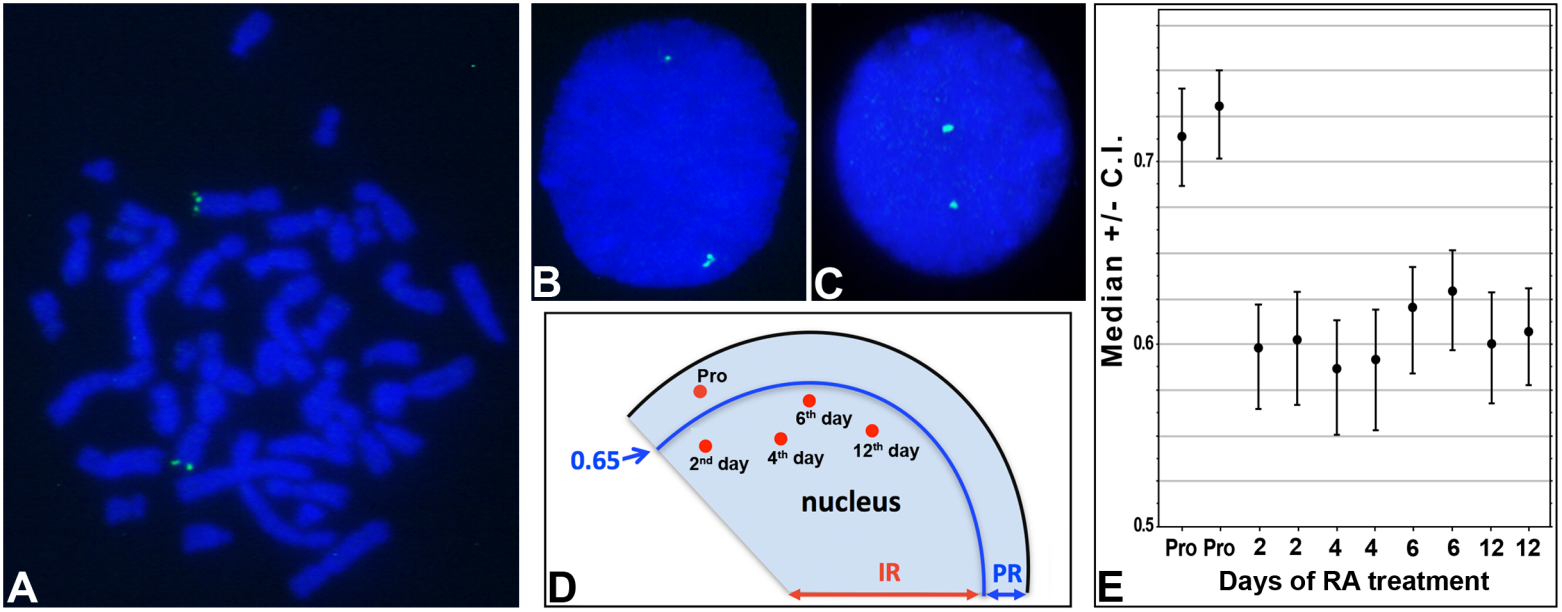
*HLXB9* gene localization in the metaphase chromosomes and interphase nuclei of SK-N-BE cells. Fluorescence *in* *situ* hybridization was used to assess localization of *HLXB9* gene, contained in the probe RPCI-5-1121A15, and visible in green in both metaphase chromosomes (A), showing localization on both chromosomes 7, and interphase nuclei (B and C). DNA is counterstained with DAPI and is visible in blue. Representative examples of proliferating (B) and differentiated SK-N-BE nuclei (C) at day 0 and 6 respectively are shown. Gene positioning in (B) is more peripheral compared to (C) as shown by the presence of green hybridization signals in different areas of the nucleus. (D) Schematic representation of *HLXB9* radial nuclear localization (red spots) in proliferating SK-N-BE (pro) cells and at the second, fourth, sixth and twelfth day after retinoic acid treatment. IR, and PR: internal and peripheral nuclear compartment respectively. 0.65 indicates the median value demarcating the peripheral and the internal nuclear compartment. (E) Median values (and the relative confidence interval, C.I.) of the data for RPCI-5-1121A15 probe located in the SK-N-BE nuclei at different days after retinoic acid treatment. Data for two different experiments were shown for each analysis. Pro indicates proliferating cells.

Using fluorescence *in* *situ* hybridization we assessed the positioning of *HLXB9* gene in the interphase nuclei of SK-N-BE cells at proliferative stage, 2^nd^, 4^th^, 6^th^, and 12^th^ day after retinoic acid induction respectively. Our data showed that *HLXB9* is localized to a more peripheral position in the proliferating SK-N-BE nuclei with median values of 0.73 whereas it occupies a more internal localization in SK-N-BE from 2^nd^ day of differentiation, with median values of 0.60. This internal location of *HLXB9* gene is conserved up to the 12^th^ day of differentiation (Figure 4).

## Discussion

The expression levels of *HLXB9* gene have been evaluated in proliferating SK-N-BE cells and at different stages of their neuronal differentiation, from day 1 to day 12 of treatment with retinoic acid. Differentiation was assessed by observing the morphological changes at the cellular level and was confirmed by real time PCR experiments through the assessment of mRNA levels of some known specific markers of differentiation such as *GAP-43*, *MAPT* and *MYCN* genes [48], [50]–[53]. Our data indicate that *HLXB9* is only expressed during the fourth, and fifth day from the start of differentiation, showing a decrease in the following days and disappearing from the ninth day. Moreover, we observed during differentiation a significant change in the *HLXB9* gene nuclear positioning, from a peripheral location in the proliferating cells to a more internal position during differentiation.

These findings indicate that the *HLXB9* gene is repositioned to the inner part of the nucleus within 2 days of differentiation to start its transcription, and then it maintains the more internal nuclear position until the end of differentiation, in a down-regulated state. Thus we can assume that *HLXB9* gene in proliferating SK-N-BE cells is maintained in a repressed status by chromatin compaction at the nuclear periphery. Then, the retinoic acid treatment seems to induce a chromatin reorganization that determines a repositioning of the genes in the nucleus, with *HLXB9* relocated in a more internal, and transcriptionally competent, compartment. After the 5^th^–6^th^ day, *HLXB9* remains in the inner part of the nucleus in a transcriptionally inactive status, probably due to the absence of specific, unidentified, transcription factor, and/or epigenetic modification of its regulatory region.

Previous studies carried out in human lymphocytes, showed that chromosome 7 is arranged in a zig-zag manner with the gene-poorest regions located close to the nuclear envelope [6]. In this context, the 7q36.3 band, where the *HLXB9* gene is mapped, has a peripheral location. This is the case also in the proliferative SK-N-BE cells, as shown in the present work. The peripheral location is associated with the absence of expression of *HLXB9*. Our findings suggest that the retinoic acid induction determines, from the first addition, a chromatin remodeling which moves the *HLXB9* gene into a transcriptionally competent compartment, toward the internal part of the nucleus. Thus, transcriptional activation of *HLXB9* could be considered a response of the SK-N-BE cells to the retinoic acid.

Although an increases in *HLXB9* expression was previously observed in motor neurons derived from human stem cells [37], our results show for the first time the change in expression level of the gene during several days of human neuronal differentiation Furthermore, *HLXB9* expression was tested in relation to its nuclear positioning in a neuronal model. Previous reports showed alterations of gene positioning within the nucleus during development. For instance, a change in the positioning of adipogenesis genes corresponding to their expression implies a reorganization of the nuclear architecture in the stem cells of pig embryos [54]. The more internal positioning of the *HLXB9* gene in the cell nucleus of neuronal cells half way through differentiation, might be crucial for its expression, making it more available to the transcription machinery. It was previously observed that a change in nuclear positioning for *HLXB9* corresponded to an increase of its transcriptional activity due to a chromosomal translocation in leukaemia [12]. Our data indicate that *HLXB9* expression is related to the chromatin reorganization not only in the case of Acute Myeloid Leukemia (AML), but also during neuronal differentiation. Being HB9 a transcription factor itself [35], [36], our findings suggest that this protein must be required only at a certain time point during neuronal differentiation, in order to initiate specific pathways conducive to cell maturity.

Our findings suggest that *HLXB9* might have a functional role in the differentiation process. Therefore, this gene could be considered a marker of development and consolidation of motor neuron, and more generally, as a marker of the early stages of neuronal differentiation, also involving a chromatin remodelling pathway, in addition to known markers that are found in completely differentiated neurons such as GAP-43, NF, MAP2, TAU [48], [50]–[53].

The possibility to use a human derived cell line has been an advantage in this work, allowing us to highlight *HLXB9* transcriptional activation in relation to the cell differentiation process and to the chromatin reorganization. Therefore, SK-N-BE cell line can be considered a good tool to study *in* *vitro* neuronal development. The use of this model enabled us also to evaluate the expression of other chromatin reorganization genes during the different stages of cell differentiation. Further work is needed to better understand the relevance of *HLXB9* in the neuronal differentiation program and to establish whether this gene could be exploited to reprogram degenerative neuronal cells. These aspects would have considerable implications in the therapy of human conditions such as Alzheimer disease.
